# Is There a Risk of Suburban Transmission of Malaria in Selangor, Malaysia?

**DOI:** 10.1371/journal.pone.0077924

**Published:** 2013-10-23

**Authors:** Kamil A. Braima, Jia-Siang Sum, Amir-Ridhwan M. Ghazali, Mustakiza Muslimin, John Jeffery, Wenn-Chyau Lee, Mohammed R. Shaker, Alaa-Eldeen M. Elamin, Ibrahim Jamaiah, Yee-Ling Lau, Mahmud Rohela, Adeeba Kamarulzaman, Frankie Sitam, Rosnida Mohd-Noh, Noraishah M. Abdul-Aziz

**Affiliations:** 1 Department of Parasitology, Faculty of Medicine, University of Malaya, Kuala Lumpur, Malaysia; 2 Department of Civil Engineering, Faculty of Engineering, University of Malaya, Kuala Lumpur, Malaysia; 3 Department of Medicine, Faculty of Medicine, University of Malaya, Kuala Lumpur, Malaysia; 4 Department of Wildlife and National Parks Peninsular Malaysia, Kuala Lumpur, Malaysia; 5 Medical Faculty, Universiti Teknologi MARA (UiTM), Sungai Buloh, Selangor, Malaysia; National University of Singapore, Singapore

## Abstract

**Background:**

The suburban transmission of malaria in Selangor, Malaysia’s most developed and populous state still remains a concern for public health in this region. Despite much successful control efforts directed at its reduction, sporadic cases, mostly brought in by foreigners have continued to occur. In addition, cases of simian malaria caused by *Plasmodium knowlesi*, some with fatal outcome have caused grave concern to health workers. The aim of this study was to investigate the possibility of local malaria transmission in suburban regions of Selangor, which are adjacent to secondary rainforests.

**Findings:**

A malaria survey spanning 7 years (2006 - 2012) was conducted in Selangor. A total of 1623 laboratory confirmed malaria cases were reported from Selangor’s nine districts. While 72.6% of these cases (1178/1623) were attributed to imported malaria (cases originating from other countries), 25.5% (414/1623) were local cases and 1.9% (31/1623) were considered as relapse and unclassified cases combined. In this study, the most prevalent infection was *P. vivax* (1239 cases, prevalence 76.3%) followed by *P. falciparum* (211, 13.0%), *P. knowlesi* (75, 4.6%), *P. malariae* (71, 4.4%) and *P. ovale* (1, 0.06%). Mixed infections comprising of *P. vivax* and *P. falciparum* were confirmed (26, 1.6%). Entomological surveys targeting the residences of malaria patients’ showed that the most commonly trapped *Anopheles* species was *An. maculatus*. No oocysts or sporozoites were found in the *An. maculatus* collected. Nevertheless, the possibility of *An. maculatus* being the malaria vector in the investigated locations was high due to its persistent occurrence in these areas.

**Conclusions:**

Malaria cases reported in this study were mostly imported cases. However the co-existence of local cases and potential *Plasmodium* spp. vectors should be cause for concern. The results of this survey reflect the need of maintaining closely monitored malaria control programs and continuous extensive malaria surveillance in Peninsula Malaysia.

## Introduction

Malaria is considered as one of the most important infectious diseases in the world. It is caused by the protozoan parasites of the genus *Plasmodium* [1]. There are an estimated 219 million malaria cases reported annually. Malaria is a potentially fatal disease. It kills approximately 660,000 people every year [2]. The vector of malaria is the *Anopheles* mosquito. Hot and humid areas are conducive for malaria transmission, where high temperature ensures the survivability of both the malarial parasites and the vectors [3,4]. In Malaysia, malaria consistently remains as a public health issue. Moreover, the infection rate have been reported to gradually increase among the mostly jungle dwelling aborigines (Orang Asli), soldiers [5], and foreign workers hailing from malaria endemic countries [6], raising concern among healthcare workers and policy makers.

Selangor is a state in Peninsula Malaysia which is highly urbanised, yet remains susceptible to malaria infections [7]. This south-western Malaysian state has an area of 7930 sq. km. It is indicated as the most populous state in Malaysia with a population of 5.46 million of which 4.99 million live in urban areas. Selangor has high level of urbanization (91.4%) and population growth [8]. Historically, Kuala Lumpur, which is the capital of Malaysia, resides in the state of Selangor before it was gazetted as Federal Territory. *Anopheles maculatus* had been shown to be an important vector of malaria in Kuala Lumpur since 1906 [9]. This was attributed to the removal of jungle cover from the valleys [3,9]. However, its present vectorial status needs to be reinvestigated.

The state of Selangor is made up of 9 districts, namely, Hulu Langat, Petaling, Kuala Selangor, Hulu Selangor, Gombak, Sabak Bernam, Klang, Kuala Langat and Sepang. Malaria has continued to persist in all these districts. In Selangor, malaria remains a healthcare concern due to various factors. Among them are human encroachment into jungles [7], high rate of construction and development [3], mobile local aborigines (Orang Asli) population [10-12] and influx of immigrant workers from malaria endemic countries [5]. Furthermore, the presence of pockets of secondary rainforests in the vicinity of urban and suburban dwellings provides niches for vector mosquitoes and monkeys that may harbor simian malaria parasites.

Recently, numerous cases of malaria caused by different species of *Plasmodium* have been reported in Selangor [5-7,13-15]. In a retrospective study conducted among the local aborigines admitted to Gombak Hospital 32 patients were positive for infection of *Plasmodium* spp [5]. (mono-infections of *Plasmodium falciparum* and *Plasmodium vivax*, mixed infections of *P. falciparum* and *P. vivax*). Reports of the simian malaria, *Plasmodium knowlesi* known to cause severe malaria in humans is also on the rise in Selangor. Recently, Lee et al reported an increase in the numbers of *P. knowlesi* malaria cases in an urban hospital [7]. Thus, there is a need to address this issue from a knowledge-based angle.

 The Geographical Information System (GIS) is a new tool that can be used to monitor and evaluate a disease and its control measures. The record of diseases can be stored and presented as maps instead of tables. The correlation between geographical factors and epidemiology provides a clearer picture of the spread of this disease [16]. This technique provides an insightful picture of the dangers of malaria. We conducted epidemiological and geographical studies in Selangor and used GIS to present the cases of malaria in the state of Selangor. Our study is timely considering the fact that the malarial transmission in Selangor needs a comprehensive study to understand its dynamics, so as to either modify the control efforts or continue the already implemented measures. Findings from this study can thus be used to support malarial eradication effort. This study attempts to understand the spread of malaria as seen in Selangor, which is a highly urbanized area. The aim of this study is to map the prevalence of malaria among the population of the state of Selangor, Malaysia, as well as to investigate the hosts and vectors of malaria in malaria endemic areas.

## Methods

### Ethics Statement

This study has been approved by Medical Ethics Committee, UMMC. Ref No. 817.18 and National Institutes of Health (NMRR-09-881-4764). Permission to review malaria cases was obtained from the Ministry of Health, and Selangor Department of Health (JKNS/KA/V/734/03-05J1D.5). Participants provided verbal informed consent. Ethics approval was given on the basis that blood taken from participants were meant for diagnostic purposes of two circumstances; the first, patient was admitted to hospital and blood was drawn to diagnose (covered by Medical Ethics Committee, UMMC. Ref No. 817.18) and the second, in areas of an outbreak or of which cases of malaria had been reported, blood samples were collected by the District Health officers after verbal informed consent had been given (covered by National Institutes of Health, NMRR-09-881-4764).

Verbal informed consent of patients in the University of Malaya Medical Centre was documented in a diagnostic form which receives final verification by the Head of the Department of Parasitology, University of Malaya (Medical Ethics Committee, UMMC. Ref No. 817.18). In areas with outbreaks or reported potential malaria cases, verbal informed consent was documented in a form which had to be filled up by the District Health Officers that contains name, age, ethnicity, gender and address of each participant. The completed forms are kept in the database of Klang Vector Laboratory (National Institutes of Health, NMRR-09-881-4764). 

This consent procedure was approved by Medical Ethics Committee, UMMC. Ref No. 817.18 and National Institutes of Health, NMRR-09-881-4764 ethics committees.

### Malaria Cases and Treatment

Malaria transmission in Selangor is sporadic and varies from one area to another. During this research project, malaria cases were detected by the health clinics of respective districts in Selangor. Blood smears were obtained and sent to the laboratory. When malaria diagnosis was confirmed, the case was registered to the source of infection (locality of infection) accordingly. The cases were confirmed by the Klang Vector Laboratory. Thereafter, the Malaria Surveillance Unit of the Klang Vector Laboratory initiates an epidemiological investigation to determine origin of the cases (whether local or imported) and conduct mosquito collections to implicate the vector. 

Treatment consisting of a single course of chloroquine was prescribed to patients with uncomplicated infections of *P. falciparum*, *P. malariae* and *P. knowlesi*. For patients infected with *P. vivax* and *P.ovale*, a course of chloroquine and primaquine was given. In cases of severe malaria, intravenous artesunate was used in combination with another drug such as doxycycline or chloroquine, regardless of the causative species. For vivax malaria patients, post treatment follow up of one year was performed whereas for patients infected with other species of malaria parasites (including *P. knowlesi* infection), a six-month-medical follow up was given. 

### Study Area and Entomological Mapping

**Figure 1:**
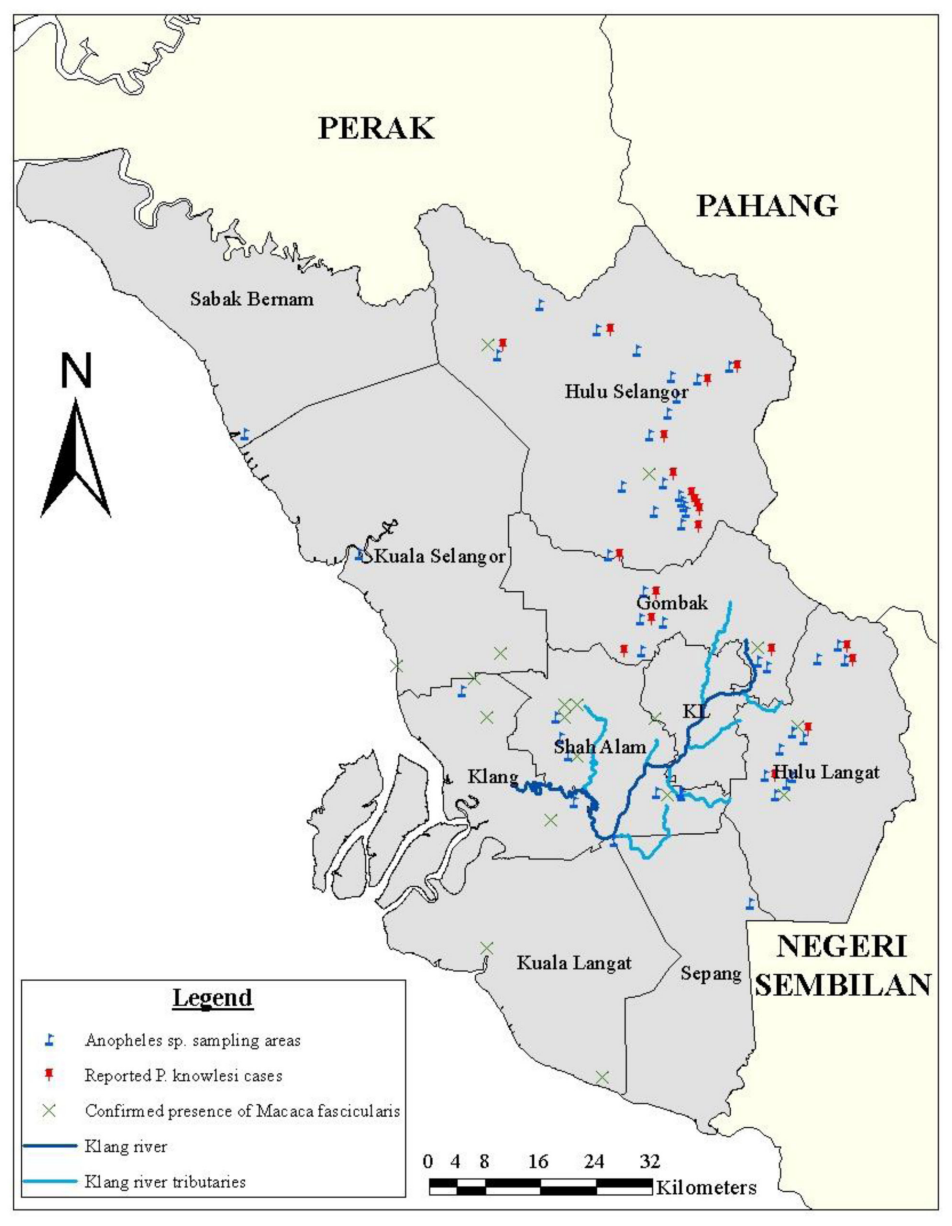
Map of the study area showing the distribution of *Anopheles* species mosquitoes, macaque monkeys, and *P.knowlesi* malaria cases. Most of Selangor districts are exposed to *Anopheles* *species* malaria vectors and *Macaca* *fascicularis*. Only Hulu Selangor has reported *P. knowlesi* cases according to vector lab records.

The state of Selangor, Peninsula Malaysia was the target site of this study. Malaria cases from all districts were reported to the Klang Vector Laboratory. Entomological sampling was done on localities with reported malaria cases between year 2006 and 2012 whereby initial investigation revealed possibility of local malarial infection. The exact coordinates of potential vectors collected were obtained using the Garmin Geographic Positioning System (GPS) and directly plotted into GIS software system [17] as in Figure 1. Larvae obtained from the surrounding vicinity were brought and raised to adults in the laboratory. Subsequently, species identification of these adult mosquitoes was performed.

### Entomological Sampling and Mosquito Identification

Sampling was done following reports of any malaria cases. Larvae were collected from water bodies adjacent (less than 1000m radius) to the vicinity where malaria cases were reported. Adult *Anopheles* mosquitoes were collected using bare leg landing method and double-bed net method for three consecutive days at 41 locations reported to have malaria cases between 2006 to 2012 (Table 1). Collection was carried out from 1800 to 0000 hours.

**Table 1:** Summary of Anopheline mosquitoes collected from different localities of human malaria cases from Selangor State.

**District**	**Locality**	**Coordinates**	**Adult Mosquito species**	**Larvae species**
Sabak Bernam	Bagan Sekinchan	3.501299 101.102279	*An.hyrcanus* group	*An.hyrcanus* group
Klang	Taman Intan, Kapar	3.16794 101.383209	*An*.*hyrcanus*group*, An.vagus*	*An.hyrcanus* group
Kuala Selangor	Jalan Raja Abdullah	3.344637 101.250488	*-*	*An.umbrosus*
Sepang	Kg. Sungai Buah, Jenderam	2.892038 101.756302	*An*.*hyrcanus*group*, An.baezai, An.karwari, An.collessi*	*An.kochi*
	Taman Mas	2.973127 101.580055	*An.hyrcanus* group	*An.hyrcanus* group
Petaling	Rumah kongsi Bandar Kinrara	3.037814 101.665731	*An. hyrcanus* group *An.karwari, An.brevipalpis*	*An. hyrcanus* group *An.karwari*
	Tmn Sri Lembayung, Sri Muda	3.02444 101.529118	*An.hyrcanus* group*, An.baezai*	*An.hyrcanus* group
	Rumah kongsi Taman Damai Utama, Bandar Kinrara	3.033041 101.668154	*An.hyrcanus* group*, An.karwari*	*An.barbirostris*
	Tmn. Pertanian Malaysia	3.107735 101.510661	*An.umbrosus*	*An.tessellatus, An.kochi*
Gombak	Rumah kongsi Myanmar dan Indonesia Hutan Bukit Rawang, Sungai Choh	3.344723 101.586786	*-*	*An. maculatus*
	Kg. Bukit Lagong	3.261972 101.628773	*An.maculatus*	*An.barbirostris*
	Tapak binaan UiTM Sg. Buloh	3.220117 101.593556	*An.maculatus, An.karwari*	*An.maculatus, An. barbirostris*
	Rumah kongsi Ukay Perdana	3.207276 101.778024	*An.maculatus*	*An.maculatus*
	Green Paradise, Templer Park	3.296599 101.63423	*An.maculatus, An.barbirostris*	*An.maculatus, An.barbirostris*
	Lembaga Perhutanan Malaysia	-	*An.aitkenii* group*, An.leucosphyrus* group *An.kochi*	*An.aitkenii* group*, An.leucosphyrus* group *An.kochi*
	Bukit Lagong	3.256617 101.642994	*An. barbirostris*	
	Bukit Lagong	3.256617 101.642994	*An. sinensis*	
Hulu Langat	Sg. Long Dalam	3.04834 101.80373	*An.hyrcanus* group *An.barbirostris*	*An*.*hyrcanus*group *An.barbirostris*
	Kg. Orang Asli Sg. Cemong	3.115834 101.813366	*An.maculatus*	*An.maculatus An.kochi An.aitkenii* group
	Taman Desa Budiman	3.057521 101.810071	*-*	*An.aitkenii* group *An.kochi*
	Kem Asli Adventure	3.222272 101.863986	*An.maculatus*	*An.maculatus, An.barbirostris*
	Sg. Lepoh, Pangsun	3.207147 101.879887	*An.maculatus*	*-*
	Hutan Rekreasi Sg. Gabai	3.210031 101.843206	*-*	*An.barbirostris, An.kochi*
	PMU Bandar Mahkota Cheras	3.059268 101.78899	*An.maculatus, An.karwari, An.aitkenii* group	*An.maculatus, An.aitkenii* group
	Sg. Serai	3.092222 101.794079	*An.karwari*	
Hulu Selangor	Kg.Orang Asli Jln Kolam Air	3.572694 101.695722	*An.leucosphyrus* group	*An.maculatus, An.kochi, An.aitkenii* group
	Restu Ibu Resort & Amberstone Ulu Yam	3.414275 101.677208	*-*	*An.maculatus, An.barbirostris*
	Ladang Getah Belakang Shell, Rasa	3.499885 101.644121	*An.brevipalpis, An.leucosphyrus* group	*An.brevipalpis*
	Rock Eco Park Resort	3.385449, 101.679324	*An.maculatus*	*An.maculatus, An.barbirostris, An.philippinensis, An.karwari*
	Ladang Getah Widuri Bukit Beruntung	3.434345, 101.589704	*An.barbirostris, An.brevipalpis, An.baezai*	*An.barbirostris*
	Kg. Orang Asli Peretak	3.59052 101.738548	*An.maculatus, An.barbirostris, An.karwari, An.baezai*	*An.maculatus*
	Hutan Simpan Bukit Belata Sg. Tengi	3.66924 101.48336	*An.umbrosus, An.leucosphyrus* group	*An.aitkenii* group*, An.kochi, An.montanus*
	Kg. Sg. Kerandang	3.576472 101.653351	*An.aitkenii* group*, An.separatus, An.baezai*	*-*
	Kg. Orang Asli Gerachi	-	*An.maculatus*	*-*
	Kalumpang Resort	3.637119 101.559835	*An.maculatus, An.leucosphyrus* group	*-*
	Kebun Buah Kolam Air Panas, Kerling	3.610993 101.609195	*An.hyrcanus* group *An.barbirostris*	*-*
	Ulu Kalong, Hulu Yam Bharu	3.421545, 101.673821	*An.maculatus*	*-*
	Ladang Getah Sg. Sendat	3.404486 101.683438	*An.maculatus, An.karwari*	*-*
	Eagle Nest Resort	3.408042 101.681807	*An.maculatus*	*-*
	Ampang Pecah	3.55013 101.66065	*An.barbirostris An.sinensis*	*-*
	Resort Taman Sri Teratai, Serendah	3.401102 101.631847	*An.karwari*	*-*
	Ampang Pecah	3.529441 101.649013	*An. sinensis*	*-*

Collected mosquitoes were identified taxonomically using *Anopheles* mosquito identification keys of Reid and Jeffery [18,19]. The dissected mosquito was examined with a Leica ICC50 microscope (Leica Microsystems, Wetzlar, Germany) equipped with a Leica DC 300F colour video camera (Leica Microsystems, Cambridge, UK), which was connected to a computer. Photographs were analysed with Leica IM500 software (Leica Microsystems, Wetzler Germany). Several organs of each mosquito specimen were investigated: midgut to check for oocysts and salivary glands for sporozoites.

### Long tailed macaque distribution

Since the long-tailed macaque monkey (*Macaca fascicularis*) is a known natural host of *P. knowlesi* [20] and is native to Peninsula Malaysia, its distribution was also taken into account in this study. Data on the macaque distribution was obtained from the Department of Wildlife and National Parks (DWNP/ PERHILITAN).

### Data analysis

Raw data of malaria patients from the Klang Vector Laboratory were archived using Microsoft Excel 2010. Data analysis was done using Sigma Plot for Windows Version 12.0 Build 12.2.0.45 and Microsoft Excel 2010 (Version 14.0.6129.500 - 32-bit). Based on the Garmin GPS Device, the coordinates of sampling points within study sites that were captured were then digitally plotted and mapped using ArcGIS software[17]. All photographs were taken with an Olympus camera (OM-D E-M5).

## Results

### Malaria cases

**Figure 2:**
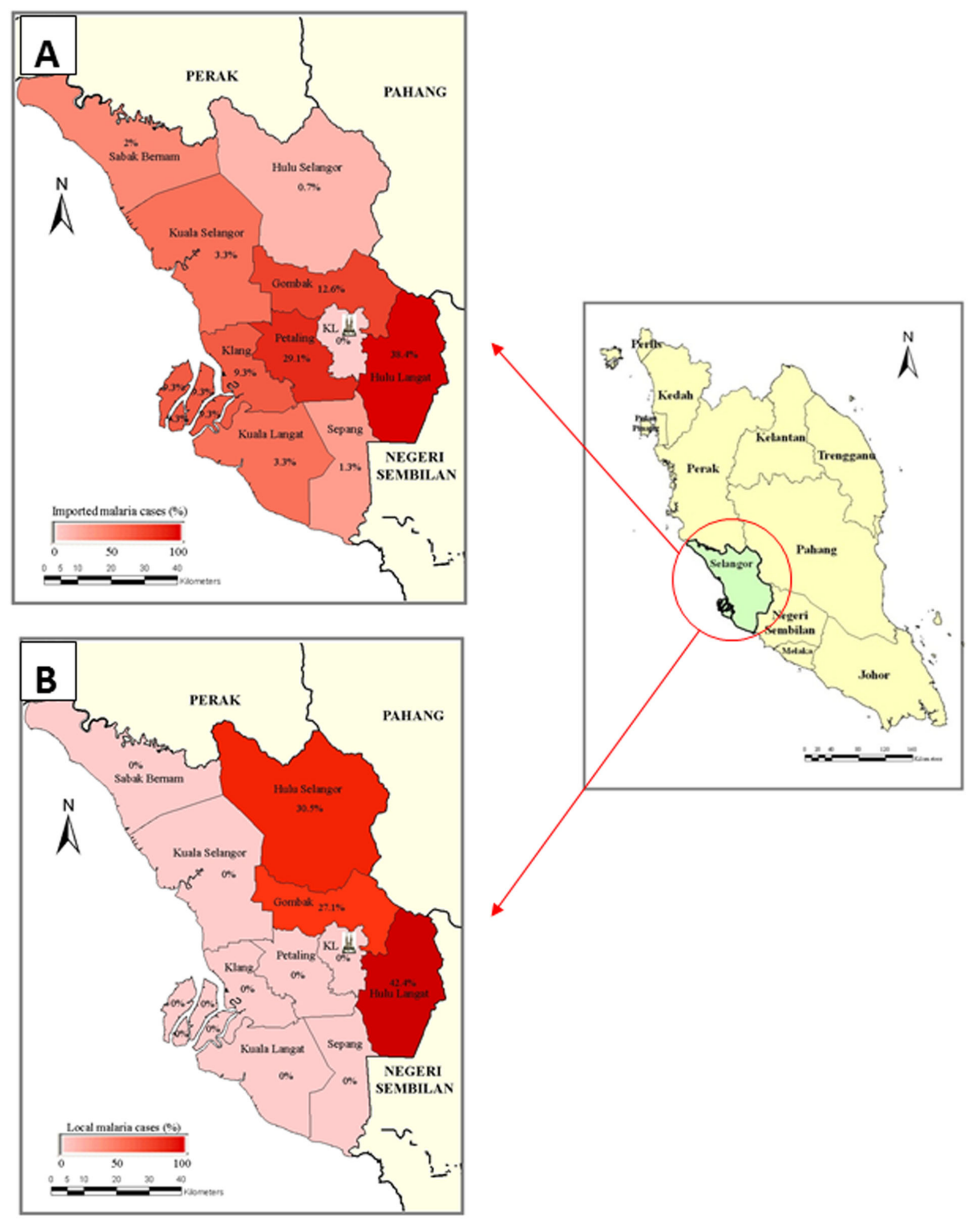
Maps of confirmed reported malaria cases by district, 2006 to 2012. Malaria endemicity showing the distribution of malaria cases, as indicated by district-level estimates based on available survey data from the Klang Vector Laboratory, Selangor, Department of Health. The districts were generated into shaded maps, in which the values for the cases are represented in shades from light (low cases) to dark (high cases). (**A**) Map showing cases that originated from outside the state of Selangor (Imported cases) (**B**). Map showing malaria cases that originated locally within the State of Selangor (Local cases).

A total of 1623 laboratory confirmed malaria cases were registered in the 7-year-period between 2006 and 2012. The malaria cases (both local and imported cases) were found positive by microscopic examination for *Plasmodium* spp. and have been reported from all the nine districts of Selangor (Figure 2). Records of confirmed positive malaria cases for the year 2006 to 2012 of each district were analysed, and percentages of malaria cases in Selangor subsequently determined, and were represented with maps accordingly. 

**Figure 3:**
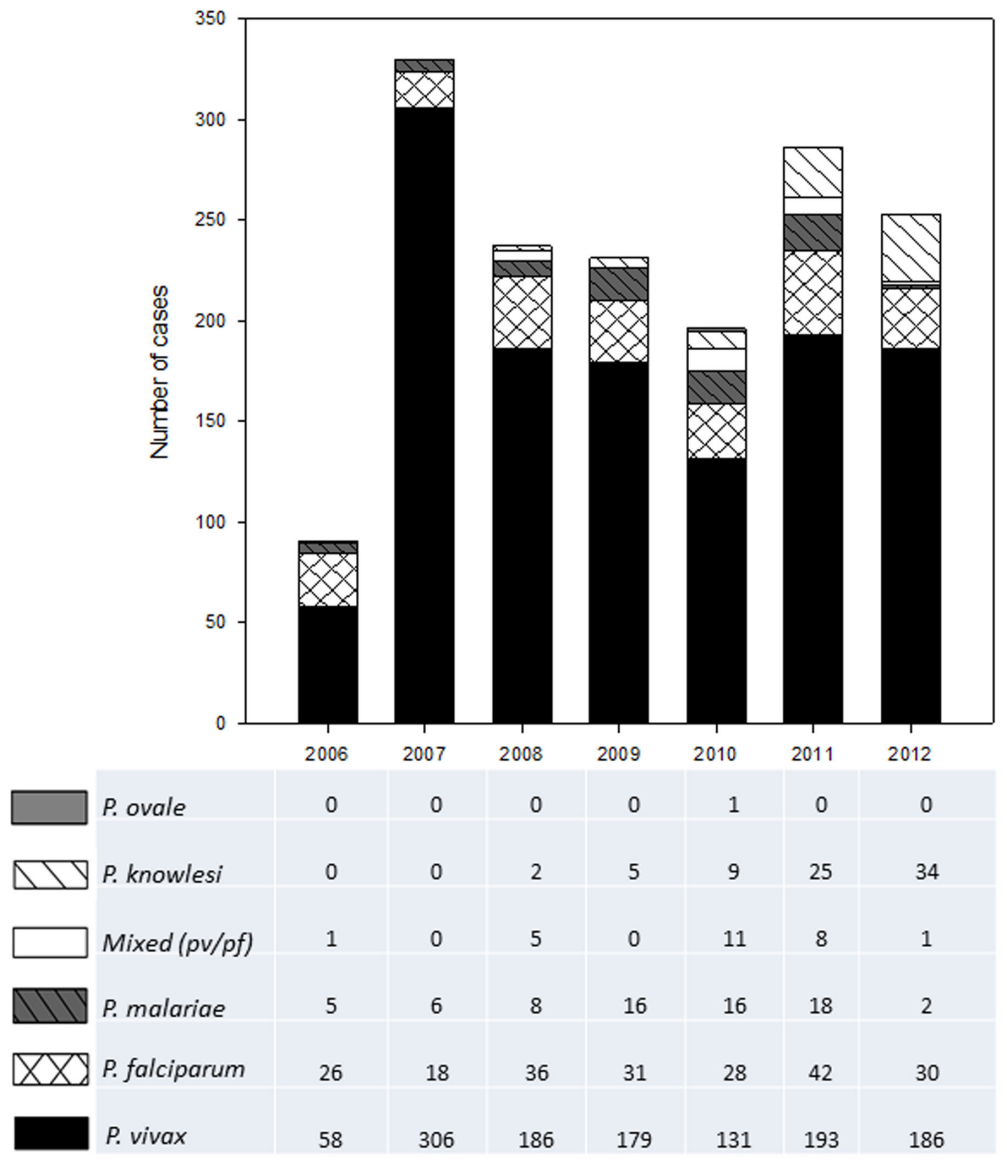
Malaria incidences caused by the different species of *Plasmodium* in Selangor (2006- 2012). Five different species of *Plasmodium* (excluding mixed cases) were identified as causes of malaria in Selangor (2006-2012). Based on the graph, from 2006 to 2012, the most common agent of malaria is *P. vivax*, followed by *P. falciparum* (with the exception of 2012, where *P. knowlesi* cases are second most common). More *P. knowlesi* cases are reported from 2008 onwards. The least common agent is *P. ovale*, with only one case in 2010. Mixed cases of *P. vivax* and *P. falciparum* are also noted in this graph.

Based on microscopic findings, of the total 1623 samples, malaria parasite with the highest prevalence was *P. vivax*, with 1239 cases (76.3%). This was followed by *P. falciparum*, with 211 cases (13.0%), *P. knowlesi*, with 75 cases (4.6%), *P. malariae*, with 71 cases (4.4%), 26 cases (1.6%) of mixed infections of *P. vivax and P. falciparum*, and 1 (0.06%) *P. ovale* infection (Figure 3).

**Figure 4:**
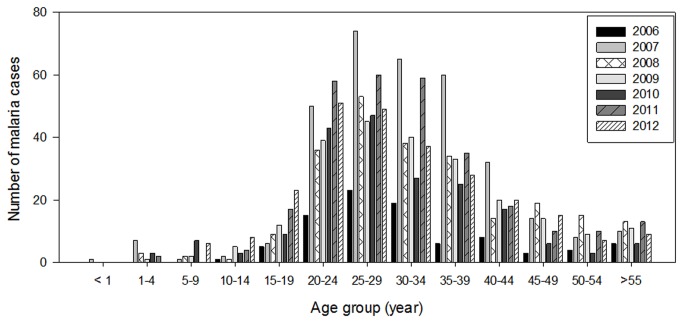
Malaria incidence in Selangor (2006-2012) based on age group. Malaria cases are seen in in all age groups, with most cases occurring in a specific group (20-39).

The age-specific infection prevalence and species distribution of all reported malaria cases are shown in Figure 4. From this study, the incidence of malaria ranged between the age of 1 and 55 year-old. The highest prevalence was found in the age group of 25- 29 year-old (peak age: 25 year-old).

**Figure 5:**
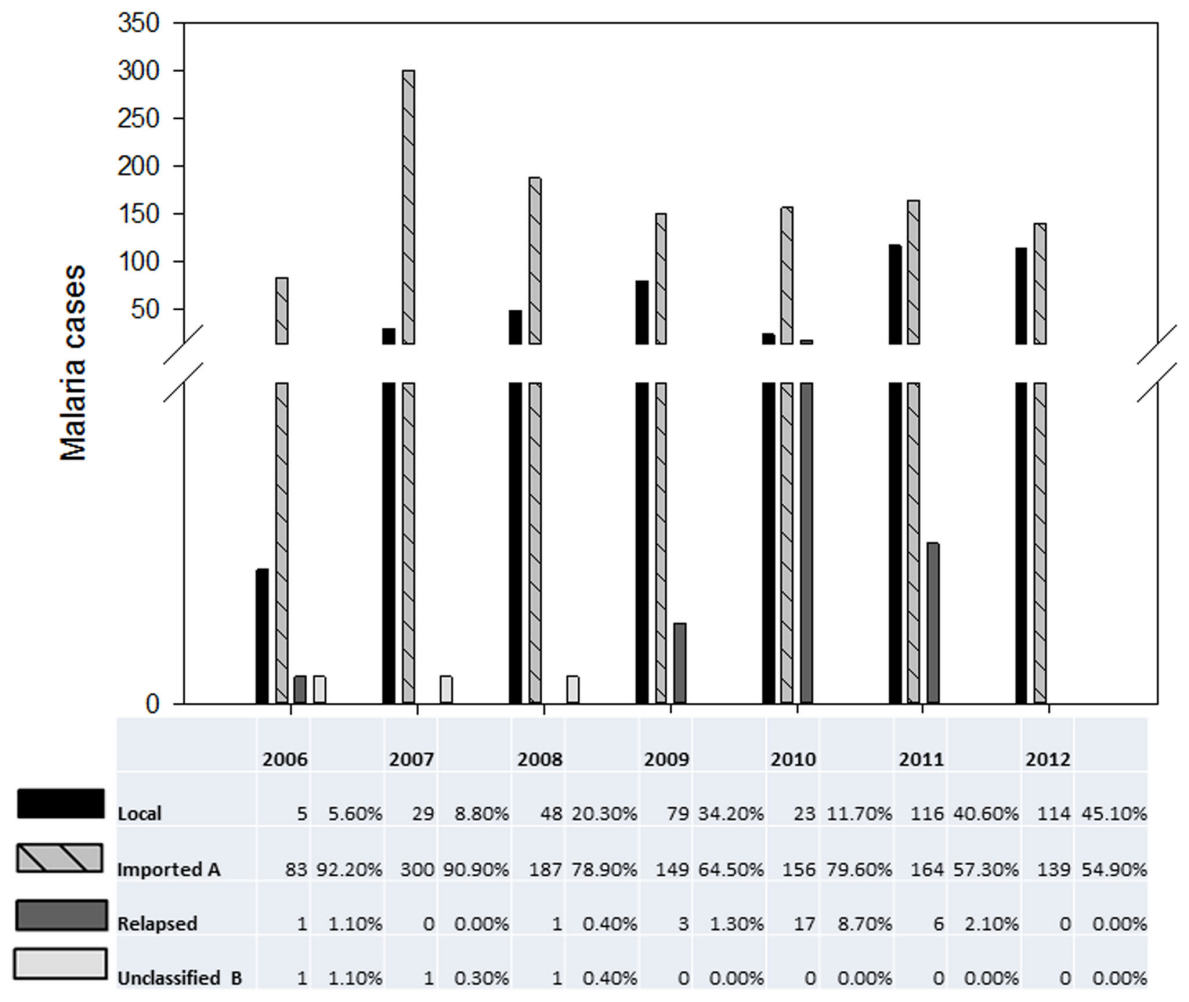
Malaria case classification in Selangor (2006-2012). Malaria cases were classified as either local, imported, relapsed cases or unclassified/unknown. Classification was based on patient history. Cases of patients residing in Selangor for 2 weeks or less would be classified as imported. As shown above, there is an increase of local cases from 2006 to 2012 (except 2010), with the highest number of local cases (116) being reported in 2011. Imported cases, though decreasing from 2007, still remain higher than local cases with the highest number of imported cases (300) being reported in 2007. By 2012, there is an almost equal percentage of imported and local cases. Relapsed cases were also reported in all years except 2007 and 2012, with 17 cases being the highest in 2010. In all relapsed cases, *Plasmodium* *vivax* was the infective agent. Some cases were denoted as unclassified due to lack of data.

Based on the distribution of imported and local malaria cases in Selangor (Figure 5), imported cases showed a sharp increase in 2007 and thereafter dropped to a level that was higher than that of year 2006. This level was generally maintained throughout the study. However, the number of local malaria cases increased. In 2012, the number of local and imported cases had almost equalised (114 local cases as compared to 139 imported cases). This is in contrast with the 5 local cases and 83 imported cases reported in 2006.

### Malaria and mosquito correlation

To determine the correlation between the causative agent of malaria (*Plasmodium*) and the vector (*Anopheles* mosquito), we tried to find out the potential vectors of malaria in the districts of Selangor with reported human cases (Figure 1; Table 1). The mosquitoes collected were taxonomically identified. Sixteen species were found, namely, the *An. hyrcanus* species group*, An. aitkenii* species group*, An. leucosphyrus* species group, *An. vagus, An. umbrosus, An. baezai, An. karwari, An. collessi, An. kochi, An. brevipalpis, An. barbirostris, An. tessellatus, An. maculatus, An. sinensis, An. philippinensis* and *An. separatus.*

In this investigation, the *Anopheles* mosquitoes were found in a total of 41 localities. The established malarial vector of Peninsula Malaysia, *An. maculatus* was relatively frequently collected in places with reported malaria cases. It was found in 18 out of the 41 locations with *Anopheles* mosquitoes. Based on examinations, none were found positive for malarial parasites. Moreover, at least 2 areas had *Macaca fascicularis* present coinciding with that of reported malaria cases and *Anopheles* mosquito prevalence (Figure 1). Both human malaria cases were that of *P. knowlesi* infections.

## Discussion

### Malaria Epidemiology and Strategy in Selangor

This study is among the few which aims to investigate the correlation between malaria cases, macaque distribution and *Anopheles* mosquitoes in Selangor, Peninsula Malaysia. The National Strategic Plan (NSP) for Malaria Elimination 2010-2020 has been initiated by the Ministry of Health Malaysia. One of the aims of this national plan is to reduce locally acquired malaria incidence rate in Peninsula Malaysia by less than 1 per 1000 population by 2015 and in East Malaysia (Sabah and Sarawak) by 2017 and eventually, to ensure that Malaysia is free from local malaria transmission (except *P.knowlesi* transmission) by 2020. However, *Anopheles* mosquitoes would still be present in Malaysia, and possibilities of outbreaks occurring in such locations still exists if malaria cases were imported from other countries. Protection in malarious areas are provided through the use of residual spraying, impregnated bed net and anti-larvicidal control methods.

One of the main obstacles faced by NSP is the mobility of infected workers. A locality that has the source of infection (human cases) is termed vulnerable areas whereas an area that has malaria vectors is categorized as receptive areas. Receptive and vulnerable areas indicate areas that have both local malaria cases being reported as well as vector mosquito being found within that malarious area simultaneously. In the event where two or more human malaria cases occur in a certain area, an “outbreak” is said to have occurred in that area. In 2012, only two out of nine districts in Selangor were classified in this category, namely Hulu Langat, and Gombak. In order to prevent outbreaks in ‘receptive and vulnerable areas’ as well as in other areas, time-tested malaria prevention and control measures of the Malaria Elimination Programme must be implemented continuously.

### Transmission of malaria in Selangor

In this study, we show that malaria transmission is still being reported in the State of Selangor. From the GIS data (Figure 2), local and imported malaria cases were still being reported from all districts of this state. It is interesting to note that although no malaria was reported within Kuala Lumpur, imported malaria transmission was reported from all the districts within the State of Selangor, which surround the Federal Territory strongly suggesting that foreigners and travellers from areas infected with malaria are crucial in the introduction of malaria to non-malarious areas [6].

On the other hand, local malaria transmission was only reported from three districts (Gombak, Hulu Langat, Hulu Selangor) that adjoins sub-urban areas and the Titiwangsa mountain range. We suggest that such distribution pattern is mainly attributed to the clearing of forests for development in these districts. Similar correlation of deforestation and malaria transmission was observed in the State of Perak, Peninsula Malaysia in 1997 [11]. 

### Factors contributing to a potential risk of malaria transmission in Selangor

#### (i) Presence of mosquito vector

We noted that *Anopheles* spp. mosquitoes were localized in areas which have undergone deforestation due to the construction of homes for human habitation in areas where secondary rainforests bordered townships [7]. A similar phenomenon has also been noted in areas of the Peruvian Amazon where malaria is on the rise due to road development and ecological changes attributed to deforestation, which in turn caused changes in the biting habits of *An. darlingi* [21,22]. In the locations studied, the larvae of *An. maculatus* in particular was found in shallow pools with clear water and plants [23]. 

Urbanised Kuala Lumpur has successfully controlled the prevalence of *Anopheles* mosquitoes by building the sub-soil pipe drainage system [24]. As a result, it is free of *An. maculatus* although having the right ecological conditions for the breeding of *An. maculatus*. Thus, it is regarded as “Maculatus Reception Areas”. This is because in the event that the drainage system built during the Malaria Eradication Programme (MEP) in the 1960s breaks down, such areas may have a sudden increase in the prevalence of *Anopheles* mosquitoes. 

Dissection performed on the *Anopheles* mosquitoes collected during the fieldwork showed that none of the specimens were infected by *Plasmodium* spp. Although *An. maculatus*, the main vector of *Plasmodium* spp. (*P. falciparum, P. malariae, P. vivax*) in Peninsula Malaysia [25] was not incriminated in this study, it is believed to be the main vector in the country and is considered to play a role as a potential vector of *P. knowlesi* due to the frequent presence of this mosquito in areas where *P. knowlesi* infection was reported in humans.

#### (ii) Presence of source of infection

Among all districts in Selangor, Petaling had the highest infection rate of malaria that was attributed to imported malaria brought in by foreign workers from malaria endemic countries. In 2012, the largest source of imported malaria was Indonesians followed by citizens of Pakistan, Myanmar, India, Bangladesh and Nepal. The two outbreaks that occurred in Hulu Langat and Gombak district in 2012 originated from the quarters of foreign workers in these districts.

### Macaque distribution

The overall data on macaque distribution in Peninsula Malaysia was obtained from the Department of Wildlife and National Parks, and detailed data on malaria cases were obtained from Health Departments of the Petaling District and Kuala Kubu Bharu District (highest imported and local cases respectively), the Klang Vector Laboratory and the University of Malaya Medical Centre (UMMC), Kuala Lumpur. *P. knowlesi* cases were seen in areas with *Macaca fascicularis* activities and mosquitoes belonging to the *Anopheles* (*Cellia*) *leucosphyrus* group (the established vector of *P. knowlesi*) had been collected from Kuala Kubu Bharu.

### Other factors

Detection and control of malaria remains a public health concern, especially among the Orang Asli who stay in remote areas and make forays into the jungle in search of jungle produce, and thus exposing themselves to Anopheline mosquitoes infected with malaria parasites. The most prevalent malaria parasite in this study was *P. vivax*, followed by *P. falciparum*. This was similar to a previous study conducted in Pos Piah, Perak by Norhayati*et al*., 2001 [26]. Other studies among Orang Asli population in Selangor reported that the most prevalent species was *P. falciparum*, followed by *P. vivax* [5]. 

Human activities such as hydroelectric dam construction, paddy field activities and irrigation canals which ecologically alter the jungle environment and encourage re-invasion of the typically efficient *Anopheles* mosquito which may play the role as vectors [11]. When a forested area is disturbed, streams and seepage water that were originally shaded are now exposed to sunlight. These clean, sun-lit water bodies are the breeding sites of choice for *An. maculatus*, the predominant mosquito vector of Peninsula Malaysia [23,25,27]. The ability of some soil types to trap water, such as those of granite origin, further contributes to this problem. Moreover, the replanting activities in agricultural industry such as those of rubber tree plantations and oil palm plantations further encourage the prevalence of the *Anopheles* mosquitoes [18,25,27]. It is possible that the construction of the dam in Kampung Pertak, Hulu Selangor district has allowed exposure of clean water bodies to sunlight, forming the breeding sites for *Anopheles* mosquitoes, such as, *An. maculatus*.

### *Plasmodium knowlesi* infection in Selangor

Over the 7-year-period, the first report of *P. knowlesi* infections (2 cases) were reported to the Klang Vector Laboratory in 2008. *P. knowlesi* cases reported in this study were mainly from jungle fringes. There were no cases reported from urban areas. In 2012, two districts reported *P. knowlesi* cases; Hulu Selangor (32 cases, 11.8%) and Gombak (5 cases, 1.8%). Both these districts had sightings of *Macaca fascicularis*, which were also verified by distribution data given by the DWNP. Prior to 2008, in this study there was no malaria reported caused by *P. knowlesi*. The discovery of a large cohort of *P. knowlesi* cases thought to have been *P. malariae* by Singh et al. [28] has led to awareness of the presence of human *knowlesi* malaria in the region. Laboratory personnel and clinicians have thus become more aware of this parasite and since then, *P. knowlesi* infections have been reported in neighbouring countries such as Thailand [29], Myanmar [30], Vietnam [31], Singapore [32], the Philippines[33], and China [34]. Besides, there were reports of *P. knowlesi* infection acquired by travellers visiting Peninsula Malaysia [35]. *P. knowlesi* infection is potentially fatal due to its rapid development (24 hour-erythrocytic cycle) and great potential of achieving high parasitemia (> 5000/μl blood) [36,37].

### Limitations

Malaria parasites species in this study were detected using light microscopy alone and thus could have led to premature analysis of results as early trophozoites that appear as ring forms resembling those of *P. falciparum* while late trophozoites “band forms” appear similar to *P. malariae* [28]. Furthermore, the malaria results provided for this study did not include infections that were not reported or those that did not go for treatment. Therefore, it may not reflect the actual malaria transmission in the state.

Another potential limitation in this study was that vegetation cover was not represented in the GIS map to show human activities such as deforestation, as well as encroachment in the sampling sites in the various districts into the potential *Anopheles* inhabited environments. However, this correlation was partially verified by our cursory field observations and findings.

Frequent immediate spraying by health authorities subsequent to a malaria case report resulted in low number of mosquitoes caught and might have led to the negative infection findings on the captured mosquitoes. Furthermore, we were limited by our capacity to enter into the depths of the jungle and had to restrict our collection close to human habitation.

## Conclusion

In line with the National Malaria Elimination Strategic Plan 2010-2020, vector control strategies consisting of residual spraying, the use of impregnated bed net and larvicidal control activities have to be done in malaria reported areas. GIS to display malaria information in a graphical representation that is easily interpreted can be extensively used in the planning of malaria control programs in all the states of Malaysia. This will be beneficial to the planning, funding and control measures that can be undertaken by the state health sector to prevent the occurrence of malaria.

Although Selangor has demonstrated a general decrease in imported malaria cases since 2007, local malaria cases, especially those caused by *P.knowlesi* are on the rise. Thus, there is still a possibility for malaria resurgence here. It is of serious concern that the presence of potential vectors in regions housing Plasmodium infected foreigners may lead to an increased risk of malaria transmission. Hence, it is imperative that the link between human populations and malaria vector should be controlled to break the transmission chain.

## References

[1] Cox-Singh J (2012) Zoonotic malaria: Plasmodium knowlesi, an emerging pathogen. Curr Opin Infect Dis 25: 530-536. doi:10.1097/QCO.0b013e3283558780. PubMed: 22710318. 22710318

[2] WHO (2012) World Malaria Report, 2012. Geneva: The World Health Organization Global Malaria Programme.

[3] Hafiz H, Nor Rasidah H (2012) Spatial and temporal distribution of malaria in Peninsular Malaysia from 1998-2010. Health Environment 3: 46-50.

[4] Garske T, Ferguson NM, Ghani AC (2013) Estimating Air Temperature and Its Influence on Malaria transmission across Africa. PLOS ONE 8: e56487. doi:10.1371/journal.pone.0056487. PubMed: 23437143. 23437143 PMC3577915

[5] Jamaiah I, Rohela M, Nissapatorn V, Mohamad Azlan H, Nor Adli A et al. (2006) A retrospective prevalence study of malaria in an aborigine hospital in Gombak, Selangor, Malaysia. Southeast Asian J Trop Med Public Health. 17547040

[6] Masitah M, Nor A, Ini M, Mas Ayu S (2008) Malaria among foreign workers in Selangor, Malaysia. The Journal of Health and Translational Medicine (JUMMEC) 11: 53-58.

[7] Vythilingam I, NoorAzian YM, Huat TC, Jiram AI, Yusri YM et al. (2008) Plasmodium knowlesi in humans, macaques and mosquitoes in peninsular Malaysia. Parasites and Vectors 1: 26. 18710577 10.1186/1756-3305-1-26 PMC2531168

[8] Department of Statistics (2010) Population Distribution and Basic Demographic Characteristics, Population and Housing Census of Malaysia, Kuala Lumpur.

[9] Hodgkin EP (1956) The Transmission of Malaria in Malaya. Studies from the Institute for Medical Research, Federated Malay States.

[10] Kaur G (2009) Malaria endemicity in an Orang Asli community in Pahang, Malaysia. Trop Biomed 26: 57-66. PubMed: 19696728. 19696728

[11] Rahman WA, Che'rus A, Ahmad AH (1997) Malaria and Anopheles mosquitos in Malaysia. Southeast Asian J Trop Med Public Health 28: 599-605. PubMed: 9561615. 9561615

[12] Martens P, Hall L (2000) Malaria on the move: Human population movement and malaria transmission. Emerg Infect Dis 6: 103–109. doi:10.3201/eid0602.000202. PubMed: 10756143. 10756143 PMC2640853

[13] Jamaiah I, Rohela M, Nissapatorn V, Khoo BL, Khoo PS et al. (2005) Malaria: a 10-year (1994-2003) retrospective study at University Malaya Medical Center (UMMC), Kuala Lumpur, Malaysia. Southeast Asian J Trop Med Public Health 36: 60-63. PubMed: 16438181. 16438181

[14] Jamaiah I, Rohela M, Nissapatorn V, Amriana A, Sumaiyah M et al. (2007) Malaria: A retrospective study in Hospital Tengku Ampuan Rahimah (HTAR), Klang, Selangor, Malaysia (2004-2006). Southeast Asian J Trop Med Public Health 38: 1-5.

[15] Lee CE, Adeeba K, Freigang G (2010) Human Plasmodium knowlesi infections in Klang Valley, Peninsula Malaysia: a case series. Med J Malays 65: 63-65. PubMed: 21265252. 21265252

[16] Lloyd O, Yu T (1994) Disease mapping: a valuable technique for environmental medicine. J-HK Med Assoc 46: 3-3.

[17] ESRI (2006) ArcGIS 9.2. Redlands, CA: Environmental Systems Research Institute.

[18] Reid J (1968) Anopheline mosquitoes of Malaya and Borneo. Studies from the Institute of Medical Research of Malaysia Bulletin No 31.

[19] Jeffery J, Rohela M, Muslimin M, Abdul Aziz SMN, Jamaiah I et al. (2012) Illustrated Keys: Some Mosquitoes of Peninsula Malaysia. University of Malaya Press. 87 pp.

[20] Chin W, Contacos PG, Collins WE, Jeter MH, Alpert E (1968) Experimental mosquito-transmission of Plasmodium knowlesi to man and monkey. Am J Trop Med Hyg 17: 355–358. PubMed: 4385130. 4385130 10.4269/ajtmh.1968.17.355

[21] Pinedo-Cancino V, Sheen P, Tarazona-Santos E, Oswald WE, Jeri C et al. (2006) Limited diversity of Anopheles darlingi in the Peruvian Amazon region of Iquitos. Am J Trop Med Hyg 75: 238-245. PubMed: 16896125. 16896125 PMC1559519

[22] Vittor AY, Pan W, Gilman RH, Tielsch J, Glass G et al. (2009) Linking deforestation to malaria in the Amazon: characterization of the breeding habitat of the principal malaria vector, Anopheles darlingi. Am J Trop Med Hyg 81: 5-12. PubMed: 19556558. 19556558 PMC3757555

[23] Ahmad R, Ali WN, Nor ZM, Ismail Z, Hadi AA et al. (2011) Mapping of mosquito breeding sites in malaria endemic areas in Pos Lenjang, Kuala Lipis, Pahang, Malaysia. Malar J 10: 1-12. doi:10.1186/1475-2875-10-1. PubMed: 21214892. 22166101 PMC3265567

[24] Singh J, Tham A (1988) Case history on malaria vector control through the application of environmental management in Malaysia.

[25] Sandosham AA, Thomas V (1983) Malariology: with special reference to Malaya. Singapore University Press.

[26] Noryahati M, Rohani AK, Hayati MN, Halimah AS, Sharom MY et al. (2001) Clinical features of malaria in Orang Asli population in Pos Piah, Malaysia. Med J Malays 56: 271-274. PubMed: 11732070. 11732070

[27] Singh J, Tham A (1988) Case history on malaria vector control through the application of environmental management in Malaysia. World Health Organization Who/VBC 88: 1-70.

[28] Singh B, Sung LK, Matusop A, Radhakrishnan A, Shamsul SS et al. (2004) A large focus of naturally acquired Plasmodium knowlesi infections in human beings. Lancet 363: 1017-1024. doi:10.1016/S0140-6736(04)15836-4. PubMed: 15051281. 15051281

[29] Jongwutiwes S, Putaporntip C, Iwasaki T, Sata T, Kanbara H (2004) Naturally acquired Plasmodium knowlesi malaria in human, Thailand. Emerg Infect Dis 10: 2211–2213. doi:10.3201/eid1012.040293. PubMed: 15663864. 15663864 PMC3323387

[30] Jiang N, Chang Q, Sun X, Lu H, Yin J et al. (2010) Co-infections with Plasmodium knowlesi and other malaria parasites, Myanmar. Emerg Infect Dis 16: 1476–1478. doi:10.3201/eid1609.100339. PubMed: 20735938. 20735938 PMC3294981

[31] Eede P, Van HN, Van Overmeir C, Vythilingam I, Duc TN et al. (2009) Human Plasmodium knowlesi infections in young children in central Vietnam. Malar J 8: 249. doi:10.1186/1475-2875-8-249. PubMed: 19878553. 19878553 PMC2773789

[32] Ng OT, Ooi EE, Lee CC, Lee PJ, Ng LC et al. (2008) Naturally acquired human Plasmodium knowlesi infection, Singapore. Emerg Infect Dis 14: 814–816. doi:10.3201/eid1405.070863. PubMed: 18439370. 18439370 PMC2600232

[33] Luchavez J, Espino F, Curameng P, Espina R, Bell D et al. (2008) Human infections with Plasmodium knowlesi, the Philippines. Emerg Infect Dis 14: 811–813. doi:10.3201/eid1405.071407. PubMed: 18439369. 18439369 PMC2600254

[34] Zhu H, Li J, Zheng H (2006) Human natural infection of Plasmodium knowlesi. Chin J Parasitol Parasit Dis 24: 70. 16866152

[35] Kantele A, Marti H, Felger I, Müller D, Jokiranta TS (2008) Monkey malaria in a European traveler returning from Malaysia. Emerg Infect Dis 14: 1434–1436. doi:10.3201/eid1409.080170. PubMed: 18760013. 18760013 PMC2603100

[36] Sinton J, Mulligan H (1932) A critical review of the literature relating to the identification of the malaria parasites recorded from monkeys of the families Cercopithecidae and Colobidae. Rec Malar Surv India: 381-443.

[37] Chin W, Contacos PG, Coatney GR, Kimball HR (1965) A naturally acquired quotidian-type malaria in man transferable to monkeys. Science 149: 865. doi:10.1126/science.149.3686.865-a. PubMed: 14332847. 14332847

